# Reports of Gastric Banding and Bowel Obstruction: A Narrative Review of the Literature

**DOI:** 10.3390/jcm13061740

**Published:** 2024-03-18

**Authors:** Antonio Vitiello, Alessandro Matarese, Giulia Sansone, Emanuela Cappiello, Giovanna Berardi, Pietro Calabrese, Roberto Peltrini, Vincenzo Pilone

**Affiliations:** 1 Advanced Biomedical Sciences Department, Naples “Federico II” University, AOU “Federico II”, Via S. Pansini 5, 80131 Naples, Italy; giovannaberardi88@gmail.com; 2 Clinical Medicine and Surgery Department, Naples “Federico II” University, AOU “Federico II”, Via S. Pansini 5, 80131 Naples, Italy; amatarese9@gmail.com (A.M.); giuliasansone97@gmail.com (G.S.); emanuela.cappiello@outlook.com (E.C.); pietro.calabres@gmail.com (P.C.); 3 Public Health Department, Naples “Federico II” University, AOU “Federico II”, Via S. Pansini 5, 80131 Naples, Italy; roberto.peltrini@unina.it (R.P.); vincenzo.pilone@unina.it (V.P.)

**Keywords:** LAGB, gastric band, occlusion, small bowel obstruction

## Abstract

The utilization rates of laparoscopic gastric banding (LAGB) declined worldwide from 42.3% in 2008 to 1.8% in 2018. Rates of complications requiring removal may reach 40–50% in the medium to long term. Bowel obstruction is a rare but severe complication that occurs after LAGB. A comprehensive literature search in PubMed was carried out to identify all available case reports of intestinal obstruction after gastric banding. The search terms were as follows: “intestinal obstruction”, “small bowel obstruction”, “gastric band”, “gastric banding”, “gastric band complications”, and “laparoscopic gastric band obstruction”. The Preferred Reporting Items for Systematic Review and Meta-Analysis (PRISMA) flowchart was used. Forty-three case reports were included in our review. Laparotomy was necessary in 18/43 (41%) of patients. Vomit was not always reported, while abdominal pain was constantly present. A CT scan was the preferred diagnostic tool. The main causes of occlusion were found to be the erosion of the gastrointestinal tract or internal hernia due to a loose tube loop. Forty-six percent of cases occurred within 5 years from insertion. Even if rare, small bowel obstruction after LAGB requires surgical intervention often with an open approach. The absence of vomit masks symptoms, but an obstruction must be always suspected in the case of severe colicky abdominal pain. A CT scan is recommended for making diagnoses.

## 1. Introduction

There is currently a worldwide epidemic of obesity. The age-adjusted prevalence of adults with a BMI > 30 kg/m^2^ in the US was 42.4% in the period between 2017 and 2018. Rates of severe obesity also increased from 4.7 to 9.2%, starting from 1999 to 2018 [1]. Similarly, according to the Italian Institute of Health, overweight and obesity rates have rapidly increased in Italy over the last few decades, as 10.2% of adults had a BMI > 30 kg/m^2^ in 2022 [2].

According to the Italian Society of Surgery for Obesity (S.I.C.OB.), 26,615 metabolic procedures were performed in Italy in 2023, and the laparoscopic adjustable gastric band (LAGB) accounted for only 4% of these interventions. The LAGB accounted for 24.4% of all weight loss interventions in 2003 and for 42.3% in 2008 [3]. However, several long-term studies demonstrated a non-response rate of up to 40–50% [4,5], and, in 2014, the percentage of bandings performed worldwide dropped to 7.4% [6]. Moreover, LAGB consists of a foreign body placed around the stomach for an undetermined period, and therefore there is some risk of severe complications, such as slippage or gastric erosion. After 10 years, the reported median long-term complication rate is 42.7% (5.9–52.9%) with a removal rate of 22.9% [7].

However, bowel obstruction, which is a well-known complication after bypass surgery [8], is rarely reported after LAGB. Diagnoses can be difficult due to the possible absence of vomit in the case of a fully inflated band. This adverse event is mostly caused by tube adhesions or band migration. A loose tube can also create a loop with subsequent internal hernia.

The aim of this review was to review the literature on case reports of small bowel obstructions after LAGB in order to analyze symptoms and clinical presentations.

## 2. Materials and Methods

A comprehensive literature search was carried out to identify all available case reports of intestinal obstruction after gastric banding. The search was performed in the PubMed electronic database. Search terms, with variations and combinations, were as follows: “intestinal obstruction”, “small bowel obstruction”, “gastric band”, “gastric banding”, “gastric band complications”, and “laparoscopic gastric band obstruction”. The Preferred Reporting Items for Systematic Review and Meta-Analysis (PRISMA) flowchart was used [9].

Case reports were included if they used patients with a history of gastric banding, presented intestinal obstruction, and were written in the English language. Case reports were excluded if they did not provide sufficient information on the clinical presentation, diagnostic methods, or outcomes of the intestinal obstruction. Two independent reviewers screened titles and abstracts for initial eligibility. Full text of potentially eligible case reports was retrieved and assessed for final inclusion. Discrepancies were resolved through discussion and consensus.

Data extraction was performed using a standardized form. The data points collected from each article were as follows: patient demographics (age and sex), a clinical presentation of intestinal obstruction, diagnostic methods, details of the gastric banding procedure, the treatment and outcomes of the intestinal obstruction, and follow-up information (when available).

## 3. Results

**Figure 1:**
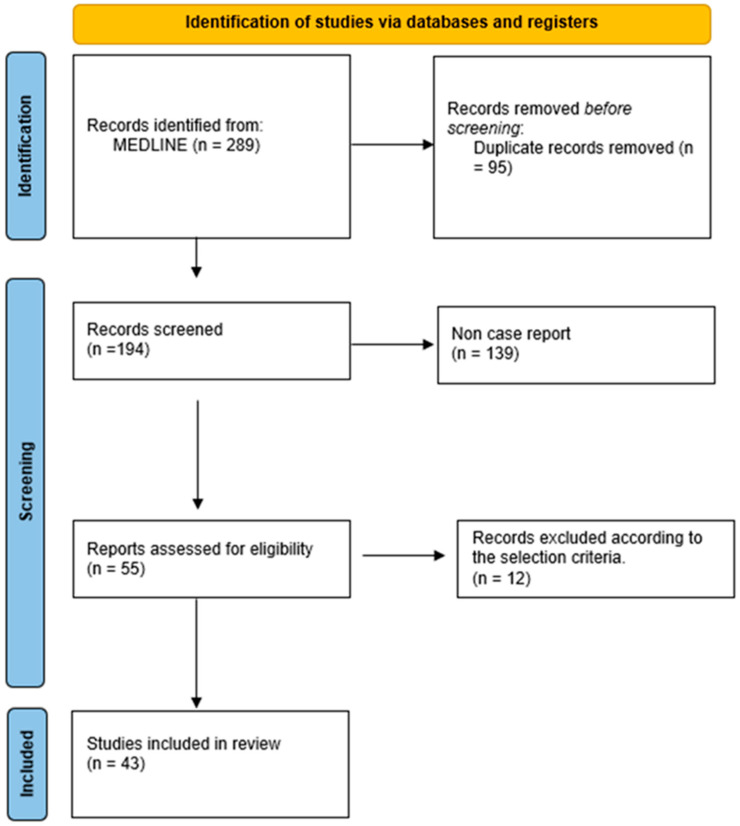
PRISMA flowchart of our search.

A total of 289 articles were found after our search. In total, 95 duplicates were removed and another 139 non-case reports were excluded. Twelve papers did not meet the selection criteria. Forty-three [10,11,12,13,14,15,16,17,18,19,20,21,22,23,24,25,26,27,28,29,30,31,32,33,34,35,36,37,38,39,40,41,42,43,44,45,46,47,48,49,50,51,52] case reports were eventually included in our review (Figure 1). The first report was published in 2000, while other articles were written last year. Before 2010, 8 out of 15 (53%) cases were treated with an open approach, while after 2010, laparotomy was necessary in 35% of cases (10/28). Vomit was reported in 26 (60%) cases, while abdominal pain was always present. A CT scan was performed in 31 (72%) patients. Migration of the silicon ring into the stomach (mostly), duodenum, jejunum or colon was the cause of the obstruction in 17 (39%) of cases, while a tube loop was responsible for the occlusion in another 17 (39%) patients. The time between band insertion and occlusion was longer than 10 years in 25% (11) of case reports, while in 20 (46%) patients, the obstruction happened in the first 5 years after LAGB (Table 1). The mean length of stay was 7.1 ± 5.4. days.

## 4. Discussion

The laparoscopic gastric band has been an important therapeutic option for patients with severe obesity for more than two decades and it has significantly contributed to the development and diffusion of bariatric surgery worldwide. The history of this procedure started in the early 1980s when Hallberg and Forsell, and, more recently, Kuzmak, developed an adjustable gastric band (AGB) device [53]. LAGB diffusion started in the mid-1990s, when laparoscopy made it possible to insert bands without open surgery [54]. However, in the last decade, the success of laparoscopic sleeve gastrectomy has caused a progressive decline in the utilization of LAGB, which has now been almost abandoned worldwide [55].

Gastric banding was originally conceived as a definitive procedure. Adjustments were meant to induce weight loss, as well as to resolve weight regain or dysphagia in order to prevent removal. Silicone was chosen due its high tolerance, which reduces the risk of abdominal adhesions [56]. However, the silicon ring is a foreign body and it may migrate or slip with time. Moreover, the port could become inflamed or infected due to multiple traumas of regulations, while the tube could cause intra-abdominal adhesions.

Nevertheless, erosion and slippage are rarely reported in large series [57,58], while unsatisfactory weight loss represents the principal reason for removal or conversion to another procedure [58,59]. Indeed, most studies have recently focused on the best secondary procedure after LAGB [20,21,22].

Our search has shown that there is a large number of case reports describing the occurrence of bowel obstruction in patients with LAGB. Therefore, the first finding of our study is that, even if rare, occlusion may happen in patients with gastric banding. This band-related complication is also severe, and it always requires surgical, or at least endoscopic, intervention. The first article of our review was published in 2000, but several studies have been reported, even in recent years. This demonstrates that, even if mostly abandoned, LAGB still represents a useful tool in the armamentarium of metabolic surgeons. Moreover, an open approach was necessary in half of the cases in the pre-laparoscopic era, but it was still required in one-third of most recent reports. In fact, occlusion due to LAGB is mostly caused by the silicon ring migrating in the gastrointestinal tract or an internal hernia migrating in a loose tube loop. In most of these conditions, a laparotomy is required to remove the band in order to assess the integrity of the bowels.

As said before, despite severe obstruction, patients with LAGB do not always vomit because the silicon ring works as a barrier against regurgitation. Subsequently, patients with LAGB and occlusion may have a severely dilated stomach under the band without important regurgitation. Therefore, it is mandatory to place a nasogastric tube in all those cases of distended and painful abdomens.

Even though the migration and erosion of the stomach (or other tracts of the gastrointestinal system) are slow processes, half of the cases were reported in the first five years after bariatric intervention [60,61]. Thus, occlusion should be suspected in the case of serious abdominal pain, even in those patients with a recent history of LAGB.

Diagnosis can be achieved with an abdominal X-ray with oral contrast, but the gold standard to find the specific cause of obstruction remains the CT scan.

Interestingly, the average postoperative recovery time after laparotomy and/or laparoscopy for occlusion in patients with LAGB is seven days. Since bariatric interventions are standardized, the mean length of stay is usually shorter, and they have acceptable 30-day morbidity and mortality rates [62,63]. Even if rare, bowel obstruction due to the gastric band is a serious condition; the migration of silicon parts into the GI tract may require intestinal or gastric resection with a subsequent need for a longer recovery period.

Even though band removals are usually performed safely and laparoscopically, surgeons should not underestimate the possible severity of occlusion which may require laparotomy and more than a week of postoperative recovery.

### Strengths and Limitations

Despite the large body of literature on LAGB, this is the first and most updated review on occlusion in patients with gastric bands to date. On the other hand, information was retrieved from case report articles with a subsequent lack of data on LAGB surgical techniques and postoperative complications after emergency surgery for occlusion.

## 5. Conclusions

There are plenty of reports on gastric banding and bowel obstruction in the literature. Even if rare, this complication requires surgical intervention, often with an open approach. The migration of the silicon ring and adhesions of the bowels to the tube system are the leading causes of occlusion after LAGB. The absence of vomit masks symptoms, but an obstruction must be always suspected in cases of severe colicky abdominal pain. A CT scan is recommended for making diagnoses.

## Figures and Tables

**Table 1:** Specifics of the case reports included in our review.

AUTOR	YEAR	AGE	SEX	BMI PRE/POST	TIME SINCE INSERTION	PRESENTATION	DIAGNOSTIC	TREATMENT	HOSPITAL STAY	TYPE OF OBSTRUCTION
M. Taskin [10]	2000	56	W	50/29	4 y	Nausea, bilious vomiting, epigastric pain	RC ABD	Laparotomy	5 d	Duodenal obstruction due to the passage of the gastric band into the lumen following gastric wall erosion
I. Pinsk [11]	2004	34	W	43/	4 y	Severe epigastric pain, postprandial vomiting	Abdominal X-ray, gastrografin	Laparoscopy		Band erosion into the stomach, causing gastric outlet obstruction
C. D. Shipkov [12]	2004	42	W	43/24.4	9 y	Acute abdomen pain, hypovolemic shock	Abdominal X-ray	Laparotomy		Obstruction due to part of the ileum strangulated by the silicon tube of the band and the abdominal wall
M. Bueter [13]	2006	65	M	43/27	13 m	Vomiting, epigastric pain	Abdominal X-ray, gastrografin, gastroscopy	Laparoscopy (band removal and jejunotomy)		Migration of the band in the lower abdomen
D. Arbell [14]	2007	30	W		4 y	Abdominal pain, no vomiting	CT scan	Laparotomy	7 d	Malrotation with the midgut volvulus
D. M. Egbeare [15]	2007	49	M		3 m	Upper abdominal pain, bilious vomiting	Abdominal X-ray, CT scan	Laparotomy	8 d	Intragastric erosion and migration to the jejunum
L. Lantsberg [16]	2007	58	W	41/29	5 y	Ability to freely eat, nausea, abdominal discomfort, abdominal pain, vomiting	CT scan	Laparoscopy	5 d	Locked gastric band located in the right lower abdomen connected to the port
Campbell NA [17]	2008	70	W	51.6/32	3 y	Nausea, left upper abdominal pain	Abdominal X-ray, CT scan, gastrografin	Gastroscopy, laparotomy	15 d	Small bowel obstruction created by the gastric band
Mills JK [18]	2008	49	W		5 y	Vague abdominal pain, distention, dysphagia	Abdominal X-ray, CT scan	Laparotomy		Small bowel obstruction created by the gastric tube
A. Agahi [19]	2009	56	W		2 y	Abdominal pain and distension, nausea, constipation	Abdominal X-ray, CT scan	Laparotomy		Obstructive bowel loop hernia due to the silicon tube
A. C. Offodile [20]	2009	30	M	60/51.7	4 y	Several postprandial epigastric pain, nausea, non-bilious emesis	CT scan	Hybrid laparoscopic and endoscopic procedure	2 d	Partial duodenal obstruction due to gastric erosion and the distal migration of the LAGB
W. Van de Water [21]	2009	50	W	41/29	4 y	Postprandial colicky abdominal pain extending to the back, nausea, vomiting	EGDS, CT scan	Laparotomy	4 d	Obstructive ileus of the duodenum and jejunum in the left upper quadrant, with the silicone connecting the tube that forms a loop around the small bowel
E. Strobos [22]	2009	44	M		1 y	Abdominal pain, matery diarreha	Abdominal X-ray, CT scan, EGDS	Laparoscopy	3 d	Small bowel obstruction due to the tubin of the gastric band
W. F. De Nino [23]	2009	48	W	46/	5 m	Intermittent crampy abdominal pain, nausea, diarrhea, dehydration	CT scan	Laparoscopy, intraoperative EGDS	7 d	Small bowel obstruction from the small bowel volvulus around the band tubing, Gram-positive chylous peritoneal fluid following LAGB
K. G. Shah [24]	2010	45	W		15 y	Epigastric abdominal pain, nausea, emesis	Abdominal ultrasonography, colangio-RM, CT scan	Laparotomy	7 d	Gastric erosion and migration into the jejunum with bowel obstruction
P. Blanc [25]	2011	38	W	43/	4 y	Dysphagia	Abdominal X-ray	Laparoscopy	4 d	Dilatation of the proximal stomach, tight adhesive band across the upper stomach from the smaller curvature to the greater curvature
Tan LB [26]	2012	47	W	59.1/28.2	5 y	Abdominal pain, profusing vomit	Abdominal X-ray, CT scan	Laparoscopy + open surgery	8 d	Small bowel internal hernia caused by the silicon tube
K. Shipman [27]	2012	36	W	45.7/31	5 y	Vomiting, diarrhoea, abdominal distension, colicky pain	ABD ultrasound, CT scan	Laparotomy		Cecal volvulus with a distended caecum, small bowel mesentry looped around the gastric band
A. Bassam [28]	2012	54	W	41.2/	8 y	Fever, periumbelical pain, swelling, colicky abdominal pain, abdominal distension	CT scan, abdominal X-ray, gastrografin	Access port removal, gastric band removal		Migration of the gastric band and its connection tube in the tansverse/descending colon and in the rectal area
T. C. Sia [29]	2013	56	M		4 y	Postprandial colicky abdominal pains, obstipation, vomiting	Abdominal X-ray, CT scan	Laparoscopy + laparotomy	3 d	Obstruction with the dilatation of the proximal small bowel, distal collapse
O. Salar [30]	2013	46	W		5 y	Nausea, colicky upper abdominal pain, fever, bilious vomiting	Abdominal X-ray, CT scan	Laparoscopy (enterotomy)		Intraluminal band migration and proximal jejunal obstruction with prominent gastric dilatation
O. H. Hamed [31]	2013	38	M	38/	3 y	Nausea, vomiting, abdominal pain	CT scan	Laparoscopy		High-grade small bowel obstruction with the transition point related to the band tubing forming a loop around the base of the mesentery of the small bowel
M. Hasemzadeh [32]	2013	42	W	35.5/27.5	19 m	Severe vomiting, abdominal pain, low-grade fever	Gastrografin, ABD ultrasonography	Laparotomy	10 d	Looping of the connecting tube around the mesentery, incarceration of the small bowel loop between the tube and the mesentery, perforation of the incarcerated small bowel loop in two places
L. Creedon [33]	2013	41	W		3 y	Colicky right upper quadrant pain extending into back	Abdominal X-ray, ultrasound scan, CT scan	Laparoscopy	10 d	The presence of the gastric band within the proximal jejunum with some dilated loops in the small bowel proximal to the band
K. Sapalidis [34]	2013	45	M		4 y			Upper gastrointestinal endoscopy		Fixing of the gastric band into the jejunum lumen, causing obstruction, symphysis, and jejuno-jejunal fistulae
S. Carandina [35]	2013	40	W	45/29	8 y	Cramping abdominal pain, constipation, nausea, vomiting	Abdominal X-ray, CT scan	Laparoscopy	5	Small bowel obstruction secondary to gastric banding migration
F. Oppliger [36]	2014	43	M		7 y	Intermittent abdominal pain, nausea	Abdominal X-ray, CT scan	Laparoscopy		High intestinal obstruction due to the gastric band connecting tube
S. Di Saverio [37]	2015	51	W	44/34.8	11 y	Sharp abdominal pain in the epigastrium, colicky in nature, dysphagia, nausea, vomiting	CT scan	Laparoscopy		Twisting of the small bowel around a strangulating band, adhesion on the port site of the reservoir (incarcerated within an incisional hernia on the same port site)
M. Connolly [38]	2015	73	W		9 y	Copious watery diarrhea, vomiting, abdominal pain	Abdominal X-ray	Laparotomy		The clear presence of the cecal volvulus with connection tubing (circumferentially wrapped around the base of thececal mesentery and intestinal lumen)
T. Hashem [39]	2016	24	M	38.1/28.2	3 y	Acute abdomen, vomiting	ABD ultrasonography	Laparotomy	3 d	Small bowel herniation between the connecting tube and the abdominal wall
J. Lemaire [40]	2016	42	W	/38	10 y	Acute abdominal pain located in the left lank, nausea, vomiting, ileus	CT scan	Upper endoscopy, laparoscopy	7 d	Jejunal obstruction due to the migration of the LAGB
K. J. L. Suter [41]	2016	52	W		2 y	Abdominal pain, vomiting, episodes of loose stools	CT scan, EGDS	Laparoscopy	5 d	Obstructive band caused by dense adhesions of the port connecting the tube to the jejunum
A. Abeysekera [42]	2017	43	W	45/31	15 y	Colicky abdominal pain, nausea, vomiting, abdominal distenzion, obstipation	RX chest, CT scan	Laparotomy	7 d	The marked dilatation of the stomach, duodenum, jejunum, and proximal ileal loops which were fluid-filled
P. Hota [43]	2017	47	W		9 y	Intermittent shortness of breath, epigastric tenderness	Abdominal X-ray, CT scan, EGDS	Laparoscopy	7 d	Intragastric band migration within the stomach cardia
F. Reche [44]	2017	36	W		11 y	Abdominal pain, fever, jaundice	CT scan	Laparotomy	29 d	Intrajejunal migration of the gastric band, peritonitis due to peroration of the stomach, duodenojejunal flexure and jejunum
D. Widmer [45]	2018	51	W	41/36.3	13 y	Abdominal pain, nausea	CT scan	Mini laparotomy (enterotomy + intestinal resection)		Endoluminal migration
B. W. Ferris [46]	2018	53	W		10 y	Epigastric pain, nausea, vomiting	ABD ultrasound, colangio-RM	Laparoscopy		Intraluminal migration of the gastric band, causing obstruction in the ampulla of Vater
H. Nasser [47]	2019	43	M	48.8/44.5	7 y	Abdominal pain, vomiting	Abdominal utrasound, ERCP	Laparoscopy, intraoperatory EGDS, laparotomy	8 d	Acute cholecystitis, choledocholithiasis, erosion of the gastric band tubing into the stomach and duodenum with secondary distortion of the major papilla
Sleiman A. [48]	2020	69	W	/31	10 y	Epigastric pain, no nausea, no vomiting	CT scan	Endoscopic	1 d	Migration up to the proximal jejunum
Alawad M. [49]	2020	39	W	50.7/29.1	12 y	Colicky abdominal pain in the epigastrium, nausea, emesis	Chest RX, gastrografin, abdominal X-ray, MRI ABD	Laparoscopy, open surgery		Downward gastric band migration, causing the dilation of stomach fundus with small erosions
Vincenty T. [50]	2022	67	W		15 y	Abdominal pain, causing nausea, vomiting, and respiratory distress	CT scan	Open surgery		Strangulation of the small bowel via the connecting tube (white arrow) with an area of ischemia
Aili A. [51]	2023	56	M		10 y	Right lower abdominal pain, nausea, vomiting	RC ABD, CT scan, intraoperative gastroscopy	Laparoscopy		Erosion of the gastric wall, intestinal obstruction, and jejunal perforation
Sharma K. [52]	2023	46	W		2 y	Abdominal pain, nausea, vomiting	CT scan	Laparoscopy, laparotomy	8 d	Small bowel obstruction as a result of the LAGB connecting tube within the mesentery

## Data Availability

Not applicable.

## References

[1] Hales C.M., Carroll M.D., Fryar C.D., Ogden C.L. (2020). Prevalence of Obesity and Severe Obesity Among Adults: United States, 2017–2018.

[2] https://www.epicentro.iss.it/passi/dati/sovrappeso.

[3] Buchwald H., Oien D.M. (2009). Metabolic/bariatric surgery worldwide 2008. Obes. Surg..

[4] Suter M., Calmes J.M., Paroz A., Giusti V. (2006). A 10-years’ experience with laparoscopic gastric banding for morbid obesity: High long-term complication and failure rates. Obes. Surg..

[5] Topart P., Becouarn G., Ritz P. (2009). One-year weight loss after primary or revisional Roux-en-Y gastric bypass for failed adjustable gastric banding. Surg. Obes. Relat. Dis..

[6] Angrisani L., Santonicola A., Iovino P., Vitiello A., Zundel N., Buchwald H., Scopinaro N. (2017). Erratum to: Bariatric surgery and endoluminal procedures: IFSO worldwide survey 2014. Obes. Surg..

[7] Shen X., Zhang X., Bi J., Yin K. (2015). Long-term complications requiring reoperations after laparoscopic adjustable gastric banding: A systematic review. Surg. Obes. Relat. Dis..

[8] Al Harakeh A.B., Kallies K.J., Borgert A.J., Kothari S.N. (2016). Bowel obstruction rates in antecolic/antegastric versus retrocolic/retrogastric Roux limb gastric bypass: A meta-analysis. Surg. Obes. Relat. Dis..

[9] Hutton B., Catalá-López F., Moher D. (2016). La extensión de la declaración PRISMA para revisiones sistemáticas que incorporan metaanálisis en red: PRISMA-NMA [The PRISMA statement extension for systematic reviews incorporating network meta-analysis: PRISMA-NMA]. Med. Clin..

[10] Taskin M., Zengin K., Unal E. (2001). Intraluminal duodenal obstruction by a gastric band following erosion. Obes. Surg..

[11] Pinsk I., Dukhno O., Levy I., Ovnat A. (2004). Gastric outlet obstruction caused by total band erosion. Obes. Surg..

[12] Shipkov C.D., Uchikov A.P., Uchikova E.H. (2004). Small bowel obstruction by the silicone tube of the gastric band. Obes. Surg..

[13] Bueter M., Thalheimer A., Meyer D., Fein M. (2006). Band erosion and passage, causing small bowel obstruction. Obes. Surg..

[14] Arbell D., Koplewitz B., Zamir G., Bala M. (2007). Midgut volvulus following laparoscopic gastric banding—A rare and dangerous situation. J. Laparoendosc. Adv. Surg. Tech..

[15] Egbeare D.M., Myers A.F., Lawrance R.J. (2008). Small bowel obstruction secondary to intragastric erosion and migration of a gastric band. J. Gastrointest. Surg..

[16] Lantsberg L., Kirshtein B., Leytzin A., Makarov V. (2008). Jejunal Obstruction caused by migrated gastric band. Obes Surg..

[17] Campbell N.A., Brown W.A., Smith A.I., Skinner S., Nottle P. (2008). Small Bowel Obstruction Creates a Closed Loop in Patients with a Laparoscopic Adjustable Gastric Band. Obes. Surg..

[18] Mills J.K., Zakon J., Hung Nguyen M. (2008). Strangulation of the small bowel mesentery and internal hernia due to the connecting tube of a gastric band. ANZ J. Surg..

[19] Agahi A., Harle R. (2009). A Serious but rare complication of laparoscopic adjustable gastric banding: Bowel obstruction due to caecal volvulus. Obes. Surg..

[20] Offodile A.C., Okafor P., Shaikh S.N., Lautz D., Thompson C.C. (2010). Duodenal obstruction due to erosion and migration of an adjustable gastric band: A novel endoscopic approach to management. Surg. Obes. Relat. Dis..

[21] van de Water W., Vogelaar F.J., Willems J.M. (2011). An unusual complication 4 years after laparoscopic adjustable banding: Jejunal obstruction due to the connecting tube. Obes Surg..

[22] Strobos E., Antanavicius G., Josloff R. (2009). Unusual complication: Small bowel obstruction caused by tubing of gastric band. Surg. Obes. Relat. Dis..

[23] DeNino W.F., Forgione P.M. (2010). Small bowel obstruction from small bowel volvulus and gram-positive peritonitis in laparoscopic adjustable gastric banding. Surg. Obes. Relat. Dis..

[24] Shah K.G., Molmenti E.P., Nicastro J. (2011). Gastric band erosion and intraluminal migration leading to biliary and small bowel obstruction: Case report and discussion. Surg. Obes. Relat. Dis..

[25] Blanc P., Delacoste F., Atger J. (2011). A rare cause of postoperative obstruction after removal of an adjustable gastric band. J. Visc. Surg..

[26] Tan L.B., So J.B., Shabbir A. (2012). Connection tubing causing small bowel obstruction and colonic erosion as a rare complication after laparoscopic gastric banding: A case report. J. Med. Case Rep..

[27] Shipman K., Bohra A., Labib M. (2012). Caecal volvulus as a rare complication of laparoscopic adjustable gastric banding. J. Surg. Case Rep..

[28] Bassam A. (2012). Unusual gastric band migration outcome: Distal small bowel obstruction and coming out per-rectum. Pan. Afr. Med. J..

[29] Sia T.C., Gatenby P., Loganathan A., Bright T. (2013). Small bowel obstruction from laparoscopic adjustable gastric banding connecting tube. ANZ J. Surg..

[30] Salar O., Waraich N., Singh R., Awan A. (2013). Gastric band erosion, infection and migration causing jejunal obstruction. BMJ Case Rep..

[31] Hamed O.H., Simpson L., LoMenzo E., Kligman M.D. (2013). Internal hernia due to adjustable gastric band tubing: Review of the literature and illustrative case video. Surg. Endosc..

[32] Hashemzadeh M., Karamirad M., Zahedi-Shoolami L. (2013). Laparoscopic adjustable gastric banding connecting tube causing small bowel obstruction and perforation. Case Rep. Surg..

[33] Creedon L., Leeder P., Awan A. (2014). Laparoscopic adjustable gastric band erosion and migration into the proximal jejunum. Surg. Obes. Relat. Dis..

[34] Sapalidis K., Liavas L., Panteli N., Kosmatopoulos E., Anastasiadis I., Charalambides S., Kesisoglou I., Tziris N. (2013). Intrajejunal migration of adjustable gastric band: A case report. Curr. Health Sci. J..

[35] Carandina S., Valenti A., Rivkine E. (2013). Small bowel obstruction secondary to gastric banding migration. Surg. Obes. Relat. Dis..

[36] Oppliger F., Wiedmaier G., León J. (2014). Acute small bowel obstruction due to the connecting tube of a gastric band. Surg. Obes. Relat. Dis..

[37] Di Saverio S., Guiducci G.M., Boschi S., Lombardi R., Biscardi A., Zanello M., Tugnoli G., Jovine E. (2015). A Challenging Misleading Diagnosis in a Patient with Suspicion of Gastric Banding Slippage and Strangulation: Diagnosis and Laparoscopic Treatment. Obes. Surg..

[38] Connolly M., Magarakis M., Narayan M. (2015). Simultaneous cecal volvulus and gastric erosion secondary to laparoscopic adjustable gastric band. Surg. Obes. Relat. Dis..

[39] Hashem T., Soliman S.M., Wagih S. (2016). Total small bowel herniation through the space between the connecting tube of gastric band and abdominal wall: A case report of a surgical emergency. Int. J. Surg. Case Rep..

[40] Lemaire J., Dewit O., Navez B. (2016). Management of a jejunal obstruction caused by the migration of a laparoscopic adjustable gastric banding. A case report. Int. J. Surg. Case Rep..

[41] Suter K.J.L., Rajasagaram N., Nottle P. (2016). Gastric band connection tube results in small bowel obstruction: An acute emergency. J. Surg. Case Rep..

[42] Abeysekera A., Lee J., Ghosh S., Hacking C. (2017). Migration of eroded laparoscopic adjustable gastric band causing small bowel obstruction and perforation. BMJ Case Rep..

[43] Hota P., Caroline D., Gupta S., Agosto O. (2017). Laparoscopic adjustable gastric band erosion with intragastric band migration: A rare but serious complication. Radiol. Case Rep..

[44] Reche F., Mancini A., Faucheron J.-L. (2017). Gastric Band Migration Causing Jejunal Obstruction: A Life-Threatening Complication. J. Gastrointest. Surg..

[45] Widmer J.D., Schade S., Muller M.K. (2018). A 13-year journey of a gastric band—Ultimate destination terminal jejunum: A case report. J. Med. Case Rep..

[46] Ferris B.W.C., Isaacs A. (2018). Biliary obstruction secondary to migrated intra-duodenal gastric band: A case report. J. Surg. Case Rep..

[47] Nasser H., Ivanics T., Leonard-Murali S., Genaw J. (2019). A case report of an adjustable gastric band erosion and migration into the jejunum resulting in biliary obstruction. Int. J. Surg. Case Rep..

[48] Sleiman A., Studer A.S., Garneau P.Y., Denis R., Magdy M., Alanazi M., Pescarus R. (2020). Case Report: Endoscopic Removal of an Eroded Gastric Band Causing Small Bowel Obstruction upon Migration into the Proximal Jejunum. Obes. Surg..

[49] Alawad M., Abukhater M., Al-Mohaimeed K. (2020). Eroded adjustable gastric band migration causing gastric obstruction and perforation in a pregnant lady. Int. J. Surg. Case Rep..

[50] Vicenty T., Zoratti V., Ravaud S., Birnbaum D.J. (2022). Small bowel obstruction after laparoscopic adjustable gastric band: The gastric band is not always the one involved. Surgery.

[51] Aili A., Li X., Abudureyimu K. (2023). Intra-jejunal migration with intestinal obstruction and perforation after gastric banding: A case report. Heliyon.

[52] Sharma K., Arfan S., Thota S.S.P., Agbasi C., Khan L., Naqvi L., Tiesenga F. (2023). Small Bowel Obstruction Secondary to Laparoscopic Adjustable Gastric Band Connecting Tube Intertwinement Within the Mesentery: A Case Report. Cureus.

[53] Kuzmak L.I. (1986). Silicone gastric banding: A simple and effective operation for morbid obesity. Contemp Surg..

[54] Catona A., Gossenberg M., La Manna A., Mussini G. (1993). Laparoscopic gastric banding: Preliminary series. Obes. Surg..

[55] Vitiello A., Berardi G., Velotti N., De Palma G.D., Musella M. (2020). Is there an indication left for gastric band? A single center experience on 178 patients with a follow-up of 10 years. Updates Surg..

[56] Beitner M.M., Ren-Fielding C.J., Fielding G.A. (2015). Reducing complications with improving gastric band design. Surg. Obes. Relat. Dis..

[57] Vitiello A., Pilone V., Ferraro L., Forestieri P. (2018). Is the Sleeve Gastrectomy Always a Better Procedure? Five-Year Results from a Retrospective Matched Case-Control Study. Obes. Surg..

[58] Clapp B., Wynn M., Martyn C., Foster C., O’dell M., Tyroch A. (2018). Long term (7 or more years) outcomes of the sleeve gastrectomy: A meta-analysis. Surg. Obes. Relat. Dis..

[59] Musella M., Berardi G., Velotti N., Schiavone V., Vitiello A. (2021). Ten-Year Results of Laparoscopic Sleeve Gastrectomy: Retrospective Matched Comparison with Laparoscopic Adjustable Gastric Banding—Is There a Significant Difference in Long Term?. Obes. Surg..

[60] Arapis K., Tammaro P., Parenti L.R., Pelletier A., Chosidow D., Kousouri M., Magnan C., Hansel B., Marmuse J. (2016). Long-term results after laparoscopic adjustable gastric banding for morbid obesity: 18-year follow-up in a single university unit. Obes. Surg..

[61] Velotti N., Vitiello A., Berardi G., Di Lauro K., Musella M. (2021). Roux-en-Y gastric bypass versus one anastomosis-mini gastric bypass as a rescue procedure following failed restrictive bariatric surgery. A systematic review of literature with metanalysis. Updat. Surg..

[62] Musella M., Bruni V., Greco F., Raffaelli M., Lucchese M., Susa A., De Luca M., Vuolo G., Manno E., Vitiello A. (2019). Conversion from laparoscopic adjustable gastric banding (LAGB) and laparoscopic sleeve gastrectomy (LSG) to one anastomosis gastric bypass (OAGB): Preliminary data from a multicenter retrospective study. Surg. Obes. Relat. Dis..

[63] Singhal R., Cardoso V.R., Wiggins T., Super J., Ludwig C., Gkoutos G.V., Mahawar K., Pędziwiatr M., Major P., Zarzycki P. (2021). 30-day morbidity and mortality of sleeve gastrectomy, Roux-en-Y gastric bypass and one anastomosis gastric bypass: A propensity score-matched analysis of the GENEVA data. Int. J. Obes..

